# Culturable Bacterial Endophytes Associated With Shrubs Growing Along the Draw-Down Zone of Lake Bogoria, Kenya: Assessment of Antifungal Potential Against *Fusarium solani* and Induction of Bean Root Rot Protection

**DOI:** 10.3389/fpls.2021.796847

**Published:** 2022-02-09

**Authors:** Priscillar Mumo Mutungi, Vitalis Wafula Wekesa, Justus Onguso, Erustus Kanga, Steve B. S. Baleba, Hamadi Iddi Boga

**Affiliations:** ^1^Institute for Biotechnology Research, Jomo Kenyatta University of Agriculture and Technology, Nairobi, Kenya; ^2^Ecological Monitoring Department, Kenya Wildlife Service, Nairobi, Kenya; ^3^Production Department, Bioline Agrosciences - Dudutech IPM Limited, Nairobi, Kenya; ^4^State Department for Wildlife, Ministry of Tourism and Wildlife, Nairobi, Kenya; ^5^Department of Evolutionary Neuroethology, Max Planck Institute for Chemical Ecology, Jena, Germany; ^6^Botany Department, Jomo Kenyatta University of Agriculture and Technology, Nairobi, Kenya

**Keywords:** Lake Bogoria, salinity, endophyte, *Bacillus megaterium*, *Enterobacter hormaechei*, biocontrol, growth promotion

## Abstract

Vascular shrubs growing along the draw-down zones of saline lakes must develop adaptive mechanisms to cope with high salinity, erratic environmental conditions, and other biotic and abiotic stresses. Microbial endophytes from plants growing in these unique environments harbor diverse metabolic and genetic profiles that play an important role in plant growth, health, and survival under stressful conditions. A variety of bacterial endophytes have been isolated from salt tolerant plants but their potential applications in agriculture have not been fully explored. To further address this gap, the present study sought to isolate culturable bacterial endophytes from shrubs growing along the draw-down zone of Lake Bogoria, a saline alkaline lake, and examined their functional characteristics and potential in the biocontrol of the bean root rot pathogen, *Fusarium solani*. We collected shrubs growing within 5 m distance from the shoreline of Lake Bogoria and isolated 69 bacterial endophytes. The endophytic bacteria were affiliated to three different phyla (*Firmicutes, Proteobacteria*, and *Actinobacteria*) with a bias in the genera, *Bacillus*, and they showed no tissue or plant specificity. All selected isolates were positive for catalase enzyme grown in 1.5 M NaCl; three isolates (B23, B19, and B53) produced indole acetic acid (IAA) and only one isolate, B23 did not solubilize phosphate on Pikovskaya agar. Isolates, B19 and B53 exhibited more than 50% of mycelial inhibition in the dual culture assay and completely inhibited the germination of *F. solani* spores in co-culture assays while two isolates, B07 and B39 had delayed fungal spore germination after an overnight incubation. All isolates were able to establish endophytic association in the roots, stems, and leaves of been seedlings in both seed soaking and drenching methods. Colonization of bean seedlings by the bacterial endophytes, B19 and B53 resulted in the biocontrol of *F. solani* in planta, reduced disease severity and incidence, and significantly increased both root and shoot biomass compared to the control. Taxonomic identification using 16S rRNA revealed that the two isolates belong to *Enterobacter hormaechei* subsp., *Xiangfangensis* and *Bacillus megaterium*. Our results demonstrate the potential use of these two isolates in the biocontrol of the bean root rot pathogen, *F. solani* and plant growth promotion.

## Introduction

Plants are sessile and therefore have to acquire all their requirements for growth, development, and resistance to both biotic and abiotic stresses within the limited ecological unit. A host of organisms and other abiotic substances are found within an ecological unit, and plants continuously interact with these substances. Soil microorganisms play an important role in these plant interactions, establishing a symbiotic relationship with the plant thereby forming the plant microbiome. These plant–microbe interactions and their putative roles have been studied (Etesami et al., 2015; Potshangbam et al., 2017) and reviewed (Andreote et al., 2014; Berg et al., 2014; Etesami and Beattie, 2018; Hassani et al., 2018). Currently, studies on the interaction of plants and microorganisms and their impacts on plant health and survival are ongoing. Association of plants and microorganisms is either mutualistic (Castrillo et al., 2017), providing and obtaining nutrients to and from the plant; commensal, assisting the plant in growth, pathogen control, and acquisition of nutrients (Bendaha and Belaouni, 2019; Omomowo and Babalola, 2019); or pathogenic (Lim et al., 2014; Wheeler et al., 2019). Plants have been hypothesized to shape their microbial associations in the rhizosphere (Bulgarelli et al., 2012). This function is mediated by the release of root exudates which attract or repel microorganisms and the physical and chemical properties of soil which determine the soil microbial communities (Shakya et al., 2013). Plant–microbe interactions are not only restricted to the rhizosphere but the phyllosphere and the endosphere also equally provide an environment for such associations (Bulgarelli et al., 2012). The endosphere represents a highly specialized and selected microbiota, which are adapted to survival in the internal plant tissues without causing disease symptoms while establishing as endophytes (Romero et al., 2014). The selection of endosphere microbiota is driven by various factors. Experiments by Wemheuer et al. (2017) on the leaves endophytic bacteria from three different grass species grown in field conditions indicated that the selection of endophytes by the plant may be functionally driven. Experiments on two different *Arabidopsis thaliana* ecotypes grown under controlled environmental conditions on soils with different geochemistry by Bulgarelli et al. (2012) concluded that the composition of root endophytes was influenced by soil type. In addition, Zúñiga et al. (2013) and Schikora et al. (2016) reported that quorum sensing ability of bacteria using the auto-inducer, N-acyl homoserine lactone in gram negative bacteria plays a key role in bacterial endophytic colonization.

Various reports have indicated the potential role of endophytic bacteria to help plants in amelioration of biotic and abiotic stresses (Rho et al., 2018). Salinity stress in plants is one of the major abiotic factors limiting growth and affecting productivity (Nia et al., 2012). Mangroves and other vascular plants and shrubs growing along the draw-down zones of saline lakes have to develop adaptive mechanisms to cope with high salinity and fluctuating environmental conditions (Harper et al., 2003). The use of microbial endophytes, one of the mechanisms employed by these plants, has been reported to be activating host plant stress response pathways in addition to the production of phytohormones and other secondary metabolites (Eaton et al., 2008; Khan et al., 2016; Li et al., 2018). Inoculation of bacterial endophytes into crop plants confers similar benefits to the plants in ameliorating the stress conditions, thereby promoting growth and productivity. For example, inoculation of tomatoes with bacterial endophytes having indole acetic acid (IAA) and catalase production capabilities (Ali A. et al., 2017) from the mangrove, *Avicennia marina* resulted in reduced effects of salinity stress and increased biomass and chlorophyll content in *Solanum lycopersicum*. Bacterial endophytes from three different halophytes by Kearl et al. (2019) were able to enhance both root and shoot growth of alfalfa in the presence of salt.

Bacterial endophytes have also been used in the biocontrol of a vast majority of plant pathogenic fungi (Card et al., 2016). Biocontrol of fungal pathogens by endophytic bacteria is, in part, linked to the production of phytohormones (Devi et al., 2017; Li et al., 2018) that help the plant to overcome the deleterious effects of the pathogen while enhancing plant growth (Mei and Flinn, 2010; Chernin and Glick, 2012) and the production of metabolites that are antagonistic to the fungi (Ludwig-Müller, 2015; Daungfu et al., 2019). The success in the use of endophytic biocontrol bacteria can be a major stride in the management of plant fungal pathogens which are a cause of reduced yields especially in common bean production, which cause estimated annual losses of 221,000 metric tons in the sub-Saharan Africa (Paparu et al., 2018). Root rot pathogens are particularly difficult to control, as the pathogen can stay in the soil for long periods and in infected plant debris as clamydospores until conditions are favorable and a host plant is grown (Conner et al., 2014). The use of agrochemicals still remains as the major tactic in the control of these pathogens, but has persistently developed resistance in addition to environmental detriments. Seed coating with fungicides has been widely used in the management of root rots, but has produced little effect in the control of these pathogens (Xu and Kim, 2014). Biocontrol of root rot pathogens using endophytic microorganisms has been cited as a noble method as the endophytes occupy the same niche with the pathogen, produce antifungal metabolites, help the plants in the acquisition of nutrients and in the priming of plant defense (Muthukumar et al., 2017). However, to date, only a couple of endophytic biocontrol agents have been registered and are commercially available for use in sustainable agriculture, thereby calling for more studies in the search and development of biological control organisms, especially the use of endophytes.

Various studies on microbial diversity from Kenyan soda Lakes have been undertaken, mainly focusing on microorganisms from the soil, water, and sediments (Mwirichia, 2009; Grant and Jones, 2016; Kambura et al., 2016; Nyakeri et al., 2018; Orwa et al., 2020). Besides, endophytic microorganisms have been isolated from several species of mangroves and other halophilic plants and no literature is available on endophytes from shrubs growing along the draw-down zones of Kenyan soda lakes. Therefore, this study focuses on the isolation of bacterial endophyte communities associated with shrub vegetation growing along the draw-down zone of Lake Bogoria, a saline-alkaline lake along Kenya's rift valley, assessing their tolerance to salinity stress *in vitro* and screening their potential for the biocontrol of *Fusarium solani* in common beans.

## Materials and Methods

### Description of the Sampling Site

Lake Bogoria is a saline-alkaline endorheic lake which lies in the half-graben basin to the east of the Kenyan Rift valley (Mulwa et al., 2010) on 0°14′25.1700″N, 36° 6′ 21.1716″ E. The Lake is believed to have been formed as a result of tectonic and volcanic activities during the Miocene era. It forms part of the Lake Bogoria National Reserve, a UNESCO world heritage site. The area is overlain by basalts, trachytes, and phonolites with volcanic soils (Mulwa et al., 2010). The region is semi-arid, with temperatures as high as 32°C and an erratic rainfall of about 500 mm per annum, spread in two seasons within a year; between March and May, representing the long rains and from October to November, representing the short rains.

### Sample Collection and Isolation of Endophytic Bacteria

Sampling was carried out in June 2016. Healthy looking vascular shrubs growing along the draw-down zone of Lake Bogoria, within 5 m from the shoreline were collected and transported to the laboratory in plastic zipper-lock bags placed in a cool box. A second set of the same plants was collected, wrapped in newspapers, labeled and pressed for later identification by a botanist at the National Museums of Kenya. Isolation was carried out within 48 h of sample collection. Different plant parts i.e., the seeds, the roots, the stems, and the leaves were separately washed thoroughly under running tap water to remove adherent soil particles and dust; then they were superficially sterilized according to the procedure by Costa et al. (2012) with minor modifications. Plant parts were sequentially immersed in 3% sodium hypochlorite with 0.01% Tween 20 for 3 min, 70% ethanol for 3 min, and then rinsed 4 times with sterile distilled water. After sterilization, the different plant parts were aseptically placed on a sterile filter paper and left to drain excess water for about 5 min under a clean bench. Confirmation of sterilization was done by spreading 100 μl of the final rinse water on a nutrient agar plate. Sterilized plant samples were cut into small pieces of about 2 cm long and soft tissues (leaves and roots) were ground into a paste with a sterile normal saline (Li et al., 2018), serially diluted ten-fold twice and 100 μl of the second dilution was plated onto freshly prepared nutrient agar plates supplemented with 0.25 M NaCl and incubated at 28°C for 3–10 days. The stem and seeds were cut into small pieces which were placed on nutrient agar plates. Emerging bacterial colonies were streaked onto fresh nutrient agar plates under the same conditions. Pure cultures were preserved in 30% glycerol and kept at −20°C for immediate use and −80°C for long-term preservation.

### Characterization of Endophytic Bacteria

Pure endophytic bacterial colonies were grouped according to colony characteristics and representatives were chosen for further analysis.

#### Assay of Exo-Enzyme Production

Amylase activity was tested using spot inoculation on a mineral starch medium agar prepared using g/l 0.5 K_2_HPO_4_, 1 KH_2_PO_4_, 1 NH_4_CL, 0.2 MgSO_4_, 5 Starch, and 20 agar PH 8.0 (Al-johani et al., 2017). Plugs of equal diameter were excised from a 24 h old plate and inoculated at the center of a freshly prepared starch agar plate. The plates were incubated at 28°C for 48 h after which grams iodine was flooded. Halo zone around the colonies indicated starch solubilization (Mishra and Behera, 2008).

Catalase reaction was tested with 3% V/V hydrogen peroxide (Reiner, 2010). A loopful of bacteria from actively growing bacterial cultures was scooped using a sterilized wooden pin, placed on a glass slide in a petri dish, and a drop of hydrogen peroxide was added to the bacterial scoop to observe immediate effervescence for catalase-positive bacteria.

Urease activity was evaluated using Stuart's urea broth containing the following in one liter: 20 g urea, 9.5 g K_2_HPO, 9.1 g KH_2_PO, 0.1 g yeast extract, and 0.01 g phenol red having the PH of 6.8. Ten milliliters of broth were distributed in test tubes and each test tube was inoculated with 10 μl of an 18 h culture. These were incubated at 35°C for 48 h and the color change to bright pink indicated a positive urease reaction (Brink, 2010).

### Plant Growth Promotion Assays

#### Phosphate Solubilization

Phosphate solubilization potential was tested according to the procedure by Goswami et al. (2014) on the selected bacterial isolates. Briefly, bacteria were spot inoculated at the center of a Pikovskaya agar plate and the plates were incubated at 27 ± 2°C for 5 days. Formation of a halo zone around the colony indicated solubilization of phosphate by the bacteria.

#### Production of Hydrogen Cyanide

Bacteria isolates were grown on a nutrient agar supplemented with 4.4 g/L of glycine (El-rahman and Shaheen, 2016). A sterile filter paper was soaked with 0.5% picric in 2% sodium carbonate and then placed on the cover of a plate. The plate was tightly sealed with parafilm and incubated at 28°C for 3 days. Change of the filter paper from yellow to brick red indicated the production of hydrogen cyanide (HCN).

#### Indole Acetic Acid Production

Bacterial cultures were grown in Tryptic soy broth (TSB) supplemented with 0.2 mg/ml L-tryptophan and grown in a shaker at 28°C for 72 h (Goswami et al., 2014). After incubation, the cultures were centrifuged at 12,000×g for 5 min. One milliliter of the supernatant was mixed with 2 ml of Salkowski reagent (0.5M FeCl_3_ in 35% HCLO_4_) in clean dry test tubes and incubated in the dark for 30 min (Orhan, 2016). The change of the solution to a reddish-brown color was an indication of the production of IAA by the respective isolate.

### NaCl Tolerance

Endophytic bacterial salt tolerance was tested using a nutrient agar supplemented with 0, 0.5, 1 and 1.5 M NaCl and bacterial growth was observed after incubation at 28°C for 3 days. Similar concentrations of NaCl were supplemented to nutrient broth and the selected bacterial isolates were grown in an incubator shaker at 28°C, 150 RPM for 48 h. The rate of growth, as turbidity, was determined with a spectrophotometer at OD_600_ with 4 replicates of each endophytic bacteria (Jiang et al., 2019).

### Molecular Characterization

Bacterial DNA was extracted using commercial Quick DNA Fungal/Bacterial miniprep Kit (Zymo Research, CA, USA) following the instructions of the manufacturer. The integrity of the extracted DNA was visualized on 0.8% agarose gel. PCR amplification targeted the nearly full length bacterial 16S rRNA gene using the universal bacterial primer set 27F, 5′-AGAGTTTGATCCTGGCTCAG-3′ and 1492R, 5′-GGTTACCTTGTTACGACCT-3′ (Li et al., 2018). The PCR reaction mix in a 30 μl final reaction volume was comprised of each primer at 1 μl (10 pmol), 1 μl endophytic bacterial DNA, 15 μl One Taq 2x master mix (Biolabs, MA, USA), and 12 μl nuclease-free water. PCR amplifications (36 cycles) was done using the following conditions: an initial denaturation for 5 min at 95°C, denaturation at 95°C for 40 s, annealing at 55°C for 45 s, and extension for 72°C for 45 s followed by a final extension at 72°C for 5 min. PCR amplicons were visualized on a 1.8% agarose gel stained with ethidium bromide. Twenty-five microliters of the amplicons were submitted to Inqaba, South Africa for bi-directional sequencing.

#### 16S rRNA Sequence Analysis

Raw nucleotide sequences were viewed trimmed and edited using Chromas Version 2.6.6 (www.technelysium.com.au/wp/chromas). Assembly of contigs, contig ambiguity correction, and detection of double pick mutations were performed using DNABaser Version 4, Heracle Biosoft (www.DnaBaser.com). EZ BioCloud identification service (Yoon et al., 2017) and BLASTn (www.ncbi.nlm.nih.gov/BLAST) search pipeline were used to compare the assembled sequences with highly homologous bacterial sequences in the databases. Gene sequences were deposited at the NCBI GenBank. Molecular phylogenetic analysis was performed using MEGA-X (Kumar et al., 2018). Sequences were aligned using ClustalW and evolutionary history of the different taxa were inferred using the Maximum likelihood method with a bootstrap consensus of 1,000 replicates. Evolutionary distance was computed using the Tamura Nei model in the number of substitutions per site with complete elimination of the missing data and gaps. There were 1,524 positions in the final data set.

### *In vitro* Antifungal Assay

#### Effect of Endophytic Bacteria on Fungal Mycelia

Based on the 16S rRNA results and literature on antifungal activities of similar endophytic bacterial strains, 9 isolates were selected for further assessment of their biocontrol activities. The bean root rot plant pathogenic, *F. solani* used in the experiment was kindly supplied by Dudutech IPM Limited, Naivasha, Kenya. The strain was earlier isolated from infected field beans. Fungal cultures were grown on a potato dextrose agar (PDA) supplemented with 200 mg/L streptomycin sulfate and incubated in the dark at 28**°**C ± 2 for 10 days prior to the experiment. Dual culture method (Xu and Kim, 2014) was used to test for the inhibition of *F. solani* mycelial growth by the endophytic bacteria. Bacterial cultures were grown on nutrient agar plates 24 h prior to the assay. A 3 mm diameter plug from a 10-day old culture plate of *F. solani* was excised and placed about 1 cm from the edge of a freshly prepared PDA plate. A loopful of the endophytic bacterial was spot inoculated at the edge on the opposite side of the fungal plug on the same plate. Control plates contained 3 mm diameter plugs of the fungus placed at the edge of the PDA plate. Plates were sealed with parafilm, incubated at 28**°**C ± 2 and observed daily for 7 days. Three independent replicates were set for each pair of test bacteria and fungus dual cultures and control plates. The effect of the endophytic bacteria on the mycelial growth was evaluated after the seventh day by measuring the radial growth of the test fungi toward the bacteria (D1) and the radial growth of the test fungi in the control plate (D2). Antagonism of the endophytic bacteria was expressed as a percentage of mycelial inhibition on the test fungi plate calculated as D2-D1/D2 x 100.

#### *In vitro* Bacterial Cell–Fungal Conidia Interaction in a Mixed Culture

The effect of endophytic bacterial cells on germination and hyphal development of *F. solani* conidia was assessed using the method proposed by Toghueo et al. (2016) with minor modifications. Briefly, test bacterial endophytes were grown in a nutrient broth overnight. One milliliter of the overnight culture was centrifuged at 3,000g for 5 min, the supernatant was discarded, and the cells were adjusted with a sterile normal saline (0.85% NaCl) to an OD_600_ of 0.5 (~10^6^ cfu/ml). *Fusarium solani* spores were harvested from a 10-day old culture grown on a PDA, filtered in 4 layers of sterile gauze, and adjusted to a concentration of 1 × 10^4^ spores/ml in a sterile potato dextrose broth (PDB) using a Neubauer Chamber. In each treatment, 100 μl of the adjusted fungal spore suspension and 40 μl of the bacterial cell suspension were added, and the mixture was gently vortexed and incubated for 4 h at 28**°**C ± 2 to optimize cell–cell contact. All samples were set in triplicate. The control consisted of 100 μl of fungal spores and 40 μl of PDB. A 10 0μl of the bacterial-fungal mixture was then plated on freshly prepared PDA plates and a sterile coverslip was placed randomly on each plate. The plates were incubated overnight at 28**°**C ± 2. The effect of the bacterial cells on *F. solani* conidia germination, germ tube growth, and mycelium development were observed by direct microscopic observation without the use of a stain at 400× magnification.

### Test for Endophytic Competence

Two methods were used to evaluate the competence of the selected bacterial endophytes in a colonizing common bean, *Phaseolus vulgaris* variety KAT B1 (yellow bean). For seed soaking procedure, the bean seeds were surface sterilized using 3% commercial bleach for 5 min followed by 70% ethanol for 3 min and 4 rinses with sterile distilled water, and air-dried for 4 h on a sterile filter paper under a clean bench (Toghueo et al., 2016). The final rinsed water was streaked on nutrient agar plates and incubated to confirm the effectiveness of sterilization. An overnight bacterial culture was centrifuged and the cells were adjusted to OD_600_ 0.5. The sterilized bean seeds were soaked in the bacterial suspension overnight, then transferred to pots containing sterilized vermiculite moistened with sterile half-strength Hoagland's solution in a growth chamber at 27**°**C ± 2 at a 12 h: 12 h light-dark cycle. At the true leaf stage, the seedlings were uprooted from the vermiculite, washed under running tap water, wrapped with a paper towel to remove excess water, and 1 g of each plant part (the leaves, the stems, and the roots) was sterilized as the field plants. Sterile parts were ground in 9 ml sterile normal saline in sterile 2 ml tubes with a micro pestle and vortexed. The supernatant was serially diluted and plated on nutrient agar plates at triplicates of each plant part.

For drenching method, the bean seeds were sterilized as above, air dried, and then transferred to a sterilized vermiculite moistened with sterile half-strength Hoagland's media. Immediately after germination, the vermiculite around the root base of each seedling was gently removed and 1 ml of an overnight culture of the bacterial suspension was adjusted to OD_600_ of 0.5 with the sterile nutrient broth. The suspension was poured at the root base and then covered with a vermiculite. Two weeks after drenching, the seedlings were removed and assessment of colonization was undertaken as for seed coating. For each of the two methods, three bean seedlings per bacterial isolate were used for re-isolation of the endophytic bacteria. Biochemical characterization of the recovered bacterial endophytes from both, seed coating and drenching, were carried out to confirm the identity with the inoculated bacteria (Etesami et al., 2015).

### *In vivo* Biocontrol of *Fusarium solani* in a Pot Experiment

Previous experiments by Whitaker and Bakker (2019) indicated differences in the dual culture inhibition assay and the detached spikelet assay using bacterial endophytes on *Fusarium graminearum*. All the nine selected endophytic bacterial isolates were assessed for their biocontrol activities in planta. The experiment was carried out in a greenhouse in pots (15 × 17 cm). The details are mentioned in the following section.

#### Preparation of Potting Mix and Introduction of the Pathogen

A mixture of forest soil and cattle manure at a ratio of 5:1, respectively, served as the potting mixture (Mutune et al., 2016). The soil-cattle manure mixture was sterilized by autoclaving for 40 min at 121°C, left to cool, and then autoclaved again before use. Sterilized and cooled potting mixture was then distributed into pots at 1 kg per pot. Moistened soil in each pot was contaminated with 5 ml of 1.5 × 10^4^ conidia/ml (Toghueo et al., 2016), mixed well, and covered with a foil paper to retain the moisture. Control pots contained soil mixed with 5 ml of distilled water with no fungal conidia. The pots were then transferred to a greenhouse and left for 3 days before planting the bean seeds.

#### Inoculation of Bean Seeds and the Experimental Setup

The common bean, *P. vulgaris* variety KAT B1 (Yellow bean) was used in this experiment. The seeds were surface sterilized by washing in 3% of sodium hypochlorite for 1 min followed by 1 min of washing in 70% ethanol, and then rinsed 5 times in sterile distilled water. Bacterial endophytes were grown for 48 h in a nutrient broth and incubated at 28**°**C ± 2 in a rotary shaker, centrifuged at 10,000 × g for 10 min, and the cells re-suspended in a normal saline solution. The cells were diluted at OD_600_ of 0.5 and the sterilized bean seeds were soaked for 24 h before sowing. The experiment was set up with 11 treatments: 9 treatments for the different bacterial endophytes in the soil contaminated with *F. solani*; *F. solani-*inoculated soil was used only as a control, and non-inoculated soil was used as a negative control. Each treatment consisted of 4 pots with 2 seeds per pot. The pots were kept in the greenhouse in a completely randomized design and the plants were watered 3 times a week until completion of the experiment after 4 weeks.

#### Assessment of *in vivo* Biocontrol Performance

A day before the completion of the experiment, the plants were watered to loosen the soil around the roots for ease of getting the root system as intact as possible. All plants were destructively sampled, removed from the pots, and washed under running tap water to remove the adhering soil, and to enable visualization of disease lesions. A 5-point disease index on a scale of 0–4 was used to rate the severity of the disease on the roots and the hypocotyl. The ratings are as follows. 0: no disease symptom; 1: slightly brown coloration (<25%); 2: moderate discoloration (25–50%); 3: dark-colored hypocotyls and roots (>50%) and 4: severe root pruning with dead or dying plants (Motallebi et al., 2015). Disease severity was assessed using the formula:


$$\begin{array}{l} \%\text{Disease severity}=\left(\Sigma \left(\text{c }\times \text{ f}\right)/\text{n}\times \text{N}\right)\times \;100 \end{array}$$

Where:

c is the disease class.
f is the frequency of disease class.
n is the number of observations.
N is the greatest value of the empirical scale used. In this case, *N* = 4.

The fresh and dry weights of the shoots and the roots of each plant was determined. Dry weights were obtained by drying the shoots and roots in an oven at 70°C for 24 h.

### Data Analysis

Plate assays were performed in triplicates. Tested parameters in pot experiments and data on anti-fungal activities and salinity tolerance were subjected to one-way analysis of variance and the means were compared using the Student Newman Kuels test. Data on endophyte re-isolation from bean plant parts were fitted on a generalized linear model with Poisson distribution. R statistical software version 2.15.4 was used for the analysis at 95% confidence interval.

## Results

### Isolation of Bacterial Endophytes

Sixty-nine bacterial cultures were isolated and purified from the leaves, stems, seeds, and roots of the eight different shrubs collected. *Solanum incanum* had the highest number of isolates while seeds from *Prosopis juliflora* gave the lowest number of isolates. Isolation from the stem tissues gave the highest number of isolates at 45.8% (31 isolates) followed by the roots (21 isolates) at 30%, the leaves at 20% (14 isolates), and then the seeds at 4% (3 isolates) (Supplementary Table 1). The isolates were grouped into morph groups based on colony characteristics; exo-enzyme (Table 1), pigment production, and source plant. The grouping gave 33 isolates, which were used for further characterization using molecular techniques.

**Table 1:** Characterization of endophytic bacterial isolates.

**Isolate ID**	**Growth at 0.5M NaCl^a^**	**Growth at 1.5M NaCl^a^**	**Amylase production^b^**	**Catalese production^b^**	**Urease production^b^**	**Idole acetic acid^b^**
B02	+ + ++	+ + +	+	–	–	–
B19	+ + +	++	+	+	–	+
B44	+ + ++	+ + +	+	+	–	–
B52	+ + ++	+ + +	+	+	–	–
B13	+ + ++	++	+	+	+	–
B14	+ + ++	++	–	+	NT	NT
B22	+ + ++	++	–	+	–	+
B32	+ + ++	+ + +	–	+	–	+
B03	+ + ++	+ + +	–	+	NT	NT
B11	+ + +	++	–	+	–	–
B36	+ + ++	–	–	+	–	–
B47	+ + +	+	–	–	NT	NT
B53	+ + +	++	+	+	–	+
B20	+ + +	+ + +	–	+	NT	NT
B16	+ + +	+	–	+	NT	NT
B21	+ + ++	+	–	+	+	–
B46	+ + +	++	+	–	NT	NT
B51	+ + +	+	–	+	–	–
B37	+	+	+	+	–	–
B42	+ + +	+ + +	–	+	+	–
B01	+ + ++	++	–	+	+	–
B04	+ + ++	+	–	+	+	–
B07	++	++	+	+	NT	NT
B35	+ + +	+	+	+	–	–
B43	–	+	–	–	–	–
B54	+ + ++	–	+	+	+	–
B56	+ + ++	+ + +	+	+	–	–
B30	+ + ++	+ + +	–	+	NT	NT
B31	+ + +		–	+	–	–
B39	+ + +	++	–	+	–	–
B08	+ + ++	–	–	+	–	–
B23	+ + +	++	+	+	+	+
B55	+	+	+	+	+	–

a *Growth response on salt concentrations: –, no growth; +, slight growth: ++, low growth: + + +, moderate growth; + + ++, full growth*.

b *Exo-enzyme production: –, no production; +, production; NT, not tested*.

### Molecular Identification of Bacterial Endophytes

**Figure 1:**
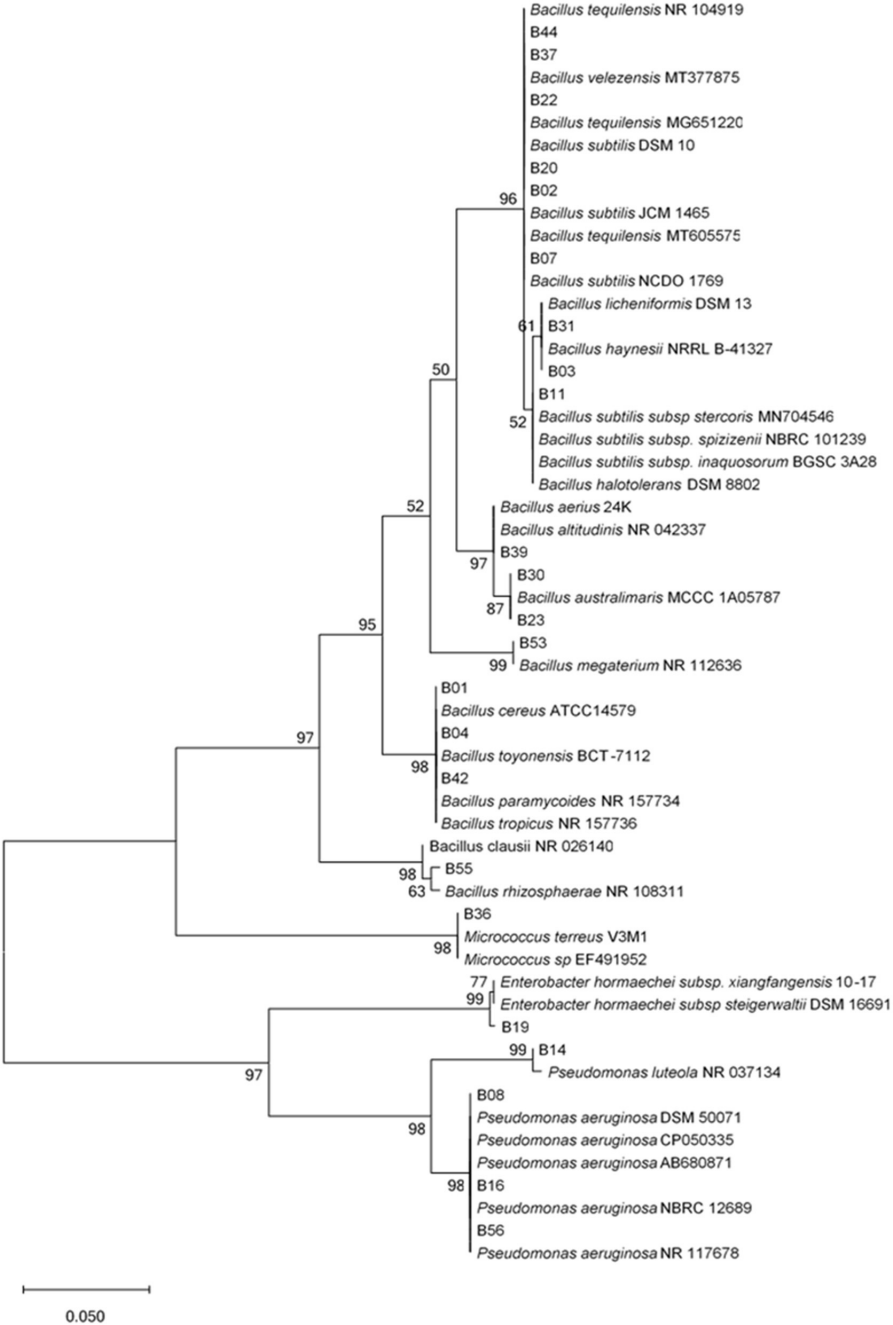
Unrooted phylogenetic tree of endophytic bacterial 16S rRNA sequences. Evolutionary history was inferred using the Maximum Likelihood method with 1,000 bootstrap replicates in MEGA X software. Scale bar represents 0.05 nucleotide substitutions per site. The percentage of trees in which the associated taxa clustered together is shown next to the branches.

Analysis of the contigs and comparison with homologous sequences in both the EZBiocloud and the National Center for Biotechnology Information (NCBI) GenBank database indicated that the isolates belonged to the following three Phyla: Firmicutes, Proteobacteria, and Actinobacteria with 99 to 100% identities to gene bank sequences (Supplementary Table 1). The Phylum, *Firmicutes* represented the highest number of isolates (26 isolates, 79%), followed by Proteobacteria (6 isolates, 18%), and Actinobacteria (I isolate, 3%) were distributed over 4 different genera: *Bacillus* (26 isolates), *Enterobacter* (1 isolate), *Pseudomonas* (5 isolates), and *Micrococcus* (1 isolate). The phylum, *Firmicutes* was represented by 11 different species isolated from all plant parts. *Bacillus subtilis* was the most frequently isolated species followed by *Bacillus velezenzis*. The Phylum, *Proteobacteria* was represented by two genera: *Enterobacter* and *Pseudomonas*. The genera, *Pseudomonas* had two species: *Pseudomonas luteola* and *Pseudomonas aeruginosa*, and were isolated from all plant parts i.e., the roots, the stems, and the leaves. The phylogenetic tree indicated two major clades (Figure 1), with the first clade comprising two subclades, one for *Actinobacteria* and the other for *Firmicutes*. The second clade comprised the *Proteobacteria* which was divided into two subclades, *Pseudomonadales* and *Enterobacterales*, confirming the identity of the bacterial endophytes.

### Enzymatic Growth Promotion Activities and Salinity Stress

**Figure 2:**
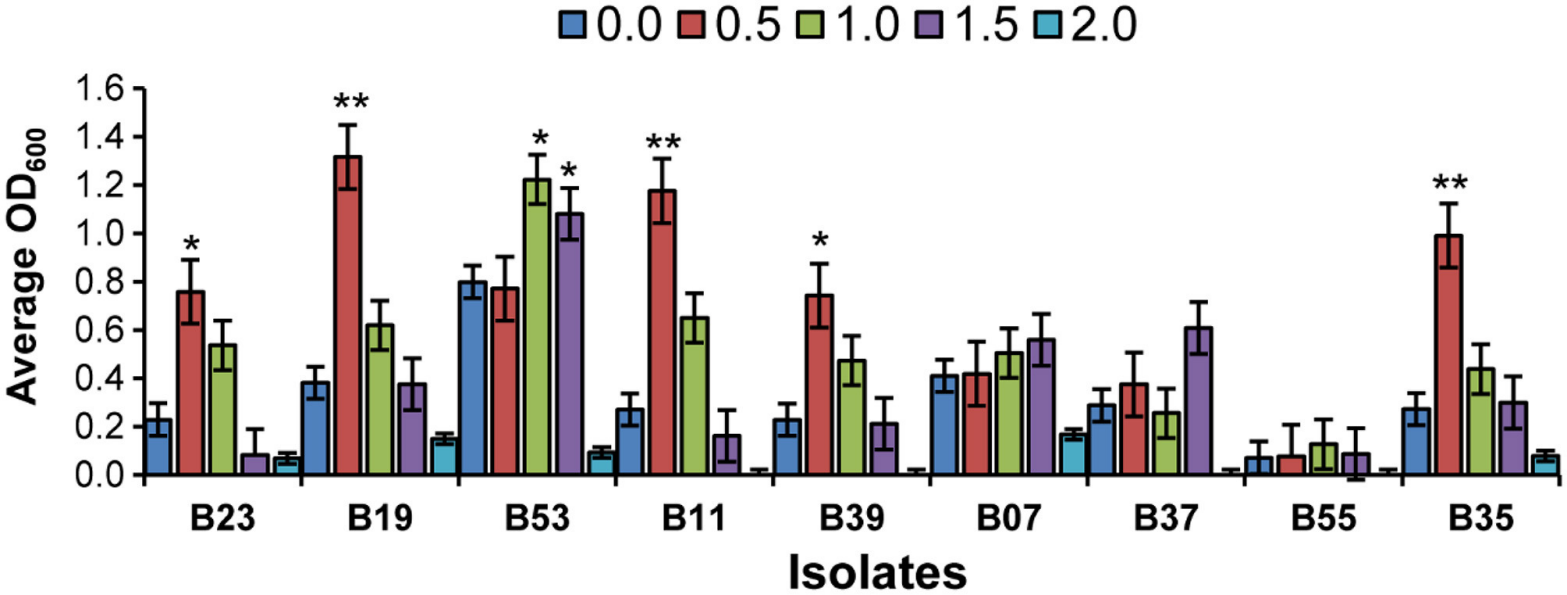
Effects of salinity stress on the selected bacteria in broth cultures at OD_600._ Bar chart represents means ± standard error (SE). Asterisk on the top of each error bar depicts significant difference of the OD_600_ among the different salt concentration in a specific isolate (One-way ANOVA: *P < 0.05, **P < 0.01).

Nine isolates were chosen based on their 16S rRNA sequence analysis and biochemical tests for further screening. The potential of the endophytic bacteria to produce amylase, catalase, urease, and IAA, were evaluated (Table 2). Only three of the isolates (B23, B19, and B53) produced IAA (Supplementary Figure 1) and one isolate, B23 was not able to solubilize phosphate while all isolates were catalase positive. The isolates, B19, B07, and B39 were the only isolates capable of producing HCN. All isolates except B39 and B11 were able to produce amylase while only three isolates, B23, B07, and B55 were positive for urease. Isolate B19 was positive for all biochemical tests except urease (Table 2). Majority of the isolates were able to grow moderately at 1.0 M NaCl. At 0.5 M NaCl, the isolates, B23, B19, B11, B39, and B35 exhibited maximum growth while the isolate, B53 exhibited maximum growth at 1.0 M NaCl (Figure 2). Only one isolate, B07 had maximum growth at 1.5 M NaCl. Isolates, B55, B37, B39, and B11 did not have any visible growth at 2.0 M NaCl and the isolate B55 exhibited the poorest growth at all tested concentrations. None of the isolates exhibited maximum growth at 0 M NaCl (Figure 2).

**Table 2:** *In vitro* growth promotion assay and biochemical tests.

**Isolates**	**Phosphate solubilisation**	**IAA**	**HCN**	**Catalase**	**Amylase**	**Urease**
B23	**–**	**+**	**–**	**+**	**+**	**+**
B37	**+**	**–**	**–**	**+**	**+**	**–**
B19	**+**	**+**	**+**	**+**	**+**	**–**
B07	**+**	**–**	**+**	**+**	**+**	**+**
B35	**+**	**–**	**–**	**+**	**+**	**–**
B53	**+**	**+**	**–**	**+**	**+**	**–**
B11	**+**	**–**	**–**	**+**	**–**	**–**
B55	**+**	**–**	**–**	**+**	**+**	**+**
B39	**+**	**–**	**+**	**+**	**–**	**–**

### Antifungal Tests

#### In vitro Antifungal Tests

**Figure 3:**
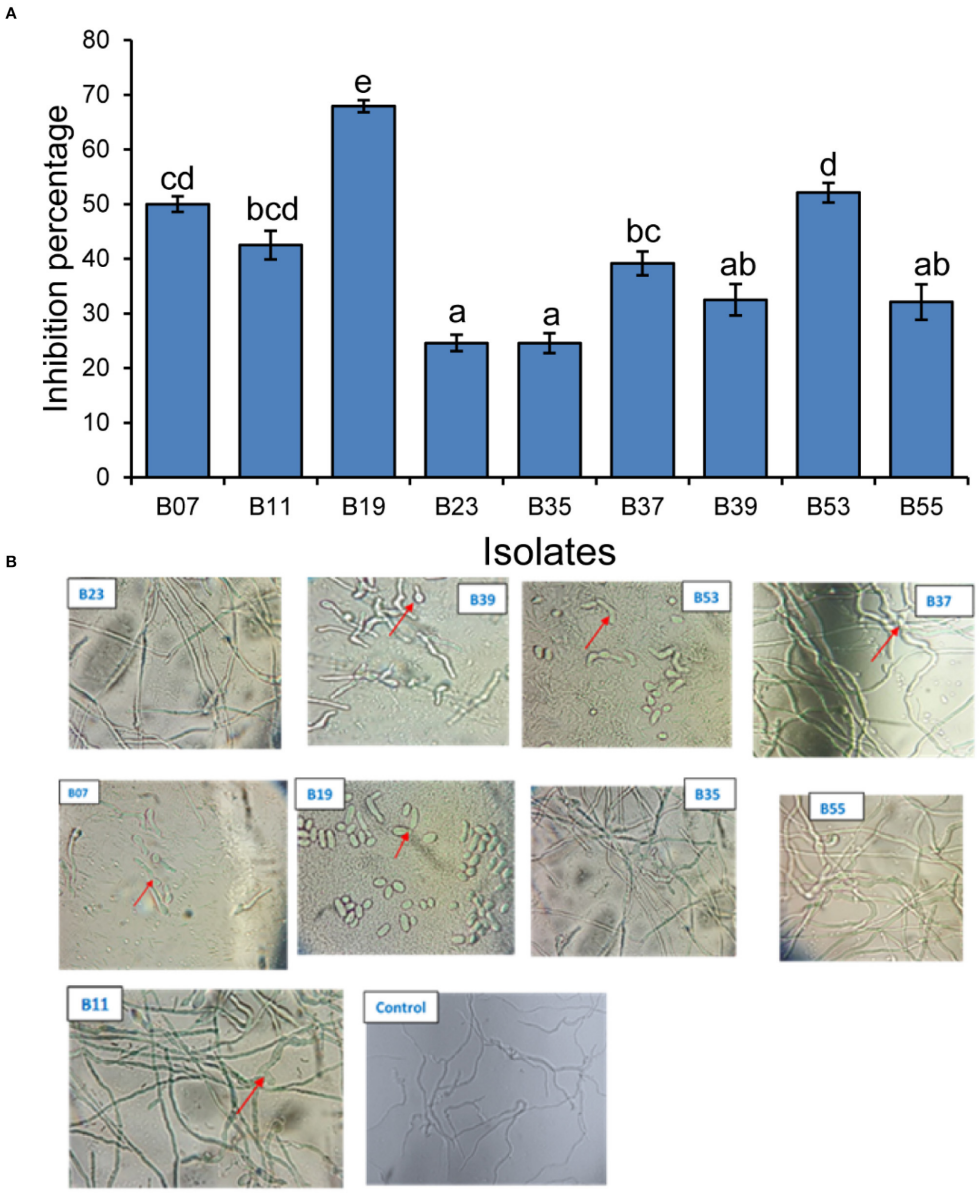
Inhibitory effect of bacterial endophytes on *Fusarium solani* mycelial and spores. **(A)** Bar plot shows the mean inhibition percentage of each isolate. Error bars represent SEM. Bars with different letters are significantly different from each other (GLM with binomial distribution followed by the analysis of deviance test, *n* = 3). **(B)** Pictures showing the effect of the interaction of *Fusarium solani* spores with bacterial endophyte cells after incubation in mixed culture plates for 24 h. Arrows in B39 and B07 indicate delayed emergence of germ tubes from spores; B53 and B19 indicate non-germinated and morphological changes in spores; B11 and B37 indicate morphological changes in the growing mycelium. Control plate contained only *F. solani* spores.

Nine bacterial isolates were selected for the evaluation of anti-fungal activities both *in vitro* and *in vivo*. The preference was guided by 16S rRNA analysis and by previous studies on the antifungal activities of similar bacterial isolates. The inhibitory activity of the isolates against *F. solani* had varying levels of inhibition of mycelial growth after incubation in a dual culture assay for 7 days as compared to the control plates (Figure 3A). Two of the isolates (B19 and B53) had the highest inhibitory activity of 67.8 and 51.9%, respectively, followed by B07 at 49.8% (Figure 3A). Isolates, B23 and B35 had the lowest mycelial inhibition rate at 24.3 and 24.3%, respectively.

Assessment of the effect of bacterial cells on *F. solani* spores was carried out in a mixed culture of fungal spores and bacterial cells. After an overnight incubation, the cells were observed under a microscope. Two isolates (B19 and B53) completely inhibited the germination of fungal spores of the pathogenic fungi upon interaction and made them appear to have undergone some morphological changes as compared to their state before incubation (Figure 3B). Isolates, B07 and B39 delayed the germination of the spores with germ tubes appearing to start emerging after overnight incubation in comparison to control plates which had a well-developed network of mycelia at the same time (Figure 3B). Isolates, B11 and B37 indicated a network of mycelia which appear to have morphological deformations unlike the straight mycelia of the control plate. Isolates, B55, B35, and B23 did not have any effect on the germination of the spores as compared to the control plate (Figure 3B).

### Endophytic Competence of Bacterial Endophytes on Beans

**Figure 4:**
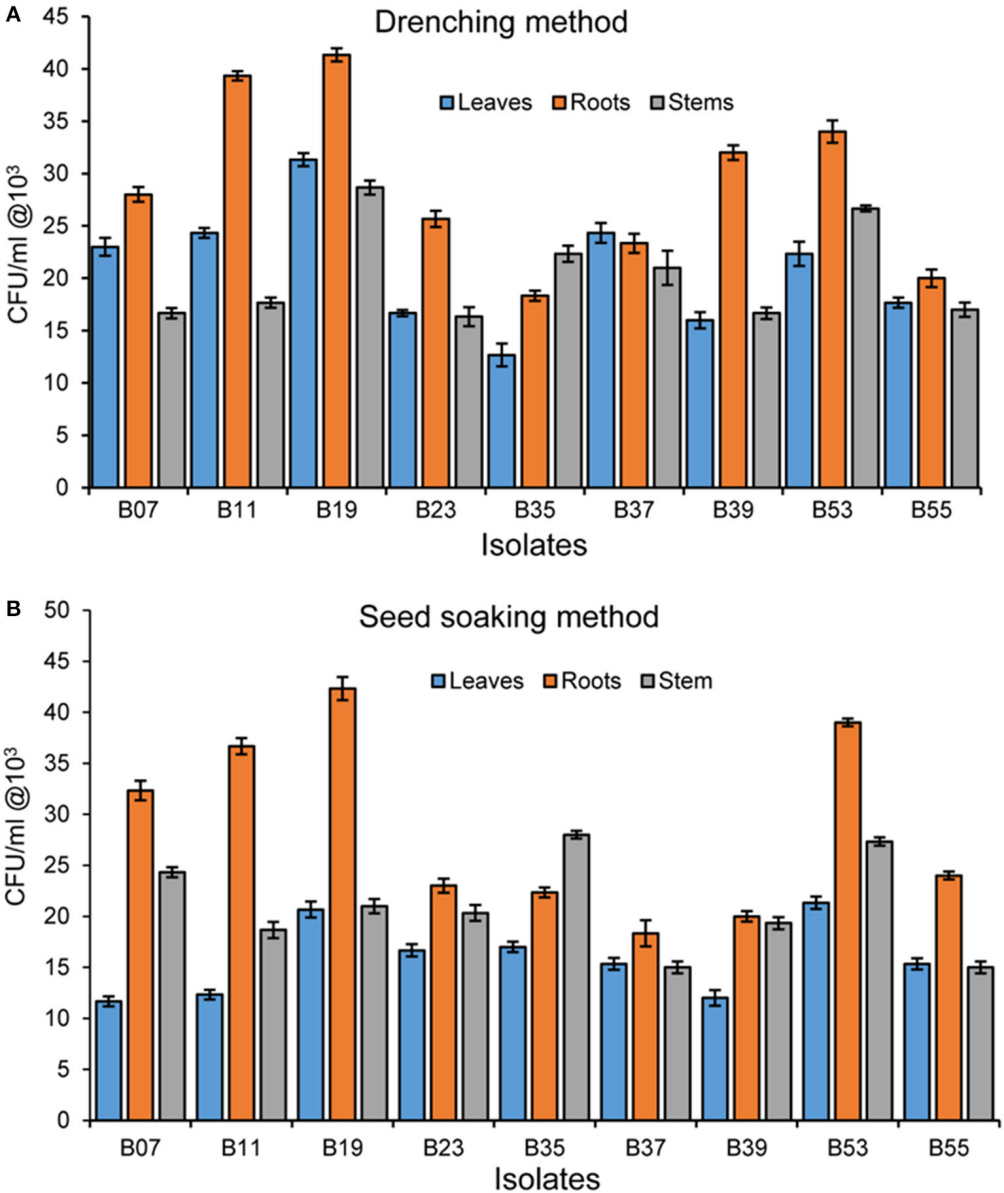
**(A)** Bacterial endophyte re-isolation from different bean plant parts following inoculation using seed soaking method. **(B)** Bacterial endophyte re-isolation from bean plant parts following inoculation using the drenching method. Bars represent SE of the mean.

To test the potential of the endophytic establishment of the isolates in beans, we used two methods; seed soaking and drenching. During re-isolation of endophytes from bean plant parts, any isolate that appeared on agar plates and that did not have similar morphological features as that of the original isolate inoculated in the seeds or applied as drenching was not counted. All tested bacterial endophytes were able to establish endophytically in all bean parts using both seed soaking and drenching methods but at different rates (Figure 4). The performance of the isolates differed significantly in the two methods (F_1, 160_ = 460.98; *p* = 0.029) and drenching gave a higher bacterial recovery titer than seed soaking. Invariably, isolate B19 had the highest re-isolation titer in all plant parts using the drenching method with 4.1 × 10^4^ cfu/g in the roots, 3.1 × 10^4^ cfu/g in the leaves, and 2.9 × 10^4^ cfu/g in the stem. It was interesting to note that isolate B35, isolated from the stem, had a higher re-isolation titer in the stems than in the leaves and roots in both the methods. Endophytic colonization was higher in the roots than in the stems and leaves. In both methods, the roots had the highest re-isolation titer at 4.2 × 10^4^ cfu/ml. For seed soaking method, isolate B39 had the lowest titer in the leaves at 1.2 × 10^4^ cfu/g.

### Biocontrol of *Fusarium solani* in a Pot Experiment

**Figure 5:**
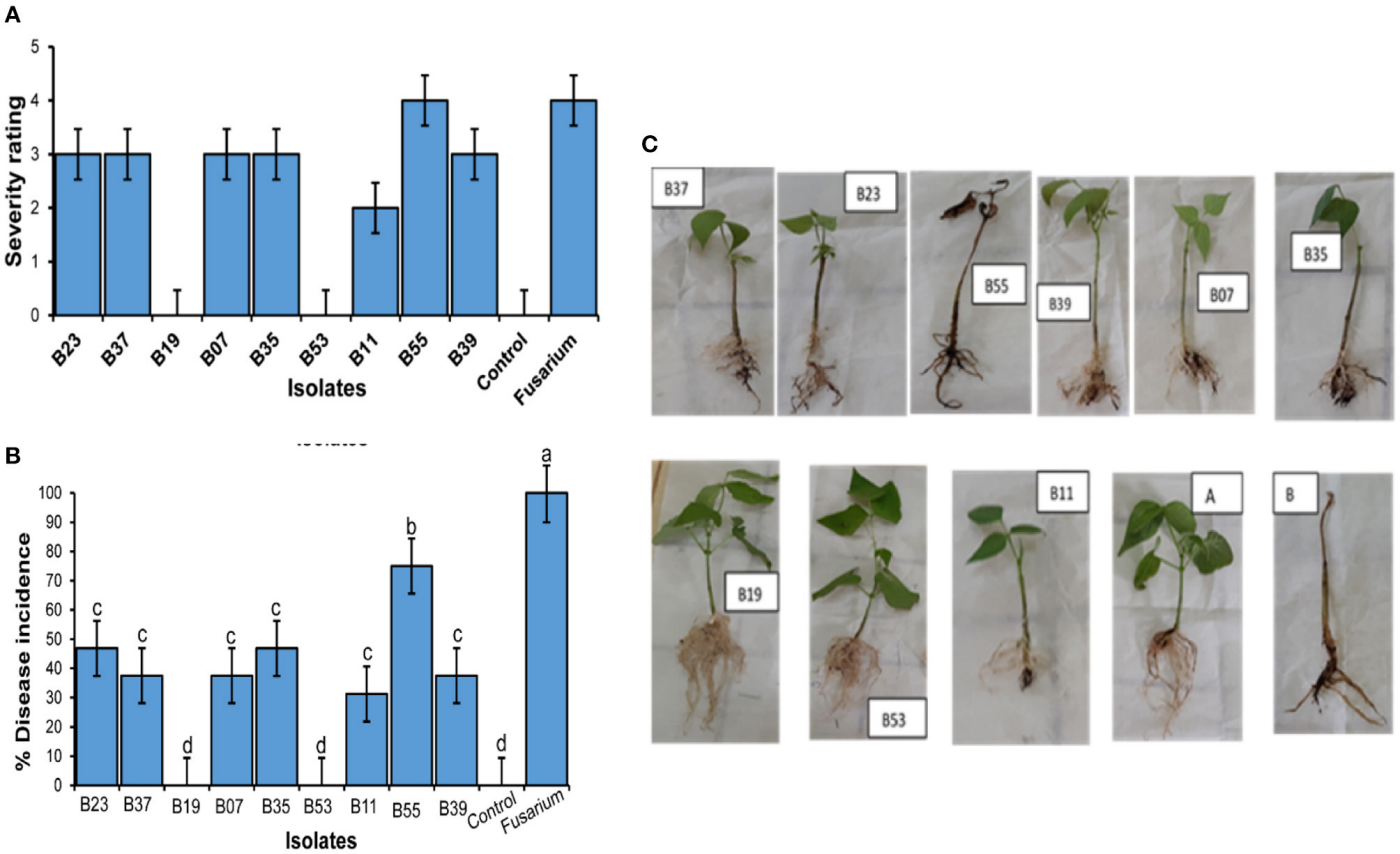
Effect of endophytic colonization of beans by bacterial endophytes on *Fusarium solani*. **(A)** Severity of symptoms in the bean root system in *Fusarium solani-*infected soil and endophytes B53 (*Bacillus megaterium*), B19 (*Enterobacter hormaechei* subsp. *Xiangfangensis*), B37 (*Bacillus tequilensis*), B07 (*Bacillus subtilis*), B35 (*Bacillus velezensis*), B11 (*Bacillus subtilis* subsp. *spezizenii)*, B39 (*Bacillus aerius*), B23 (*Bacillus australimaris*) and B55 (*Alkalihalobacillus clausii*). **(A)** Represents control seedling without endophyte and grown in un-infested soil. **(B)** Represents control seedlings without endophytic bacteria and grown in *Fusarium solani* infested soil. All plants were grown in a greenhouse for 30 days. **(B)** Percentage (±SEM) of disease incidence. Bars with different letters indicate significant difference (ANOVA, then SNK *post-hoc, p* < 0.05). **(C)** Severity rating of diseases (±SEM).

**Figure 6:**
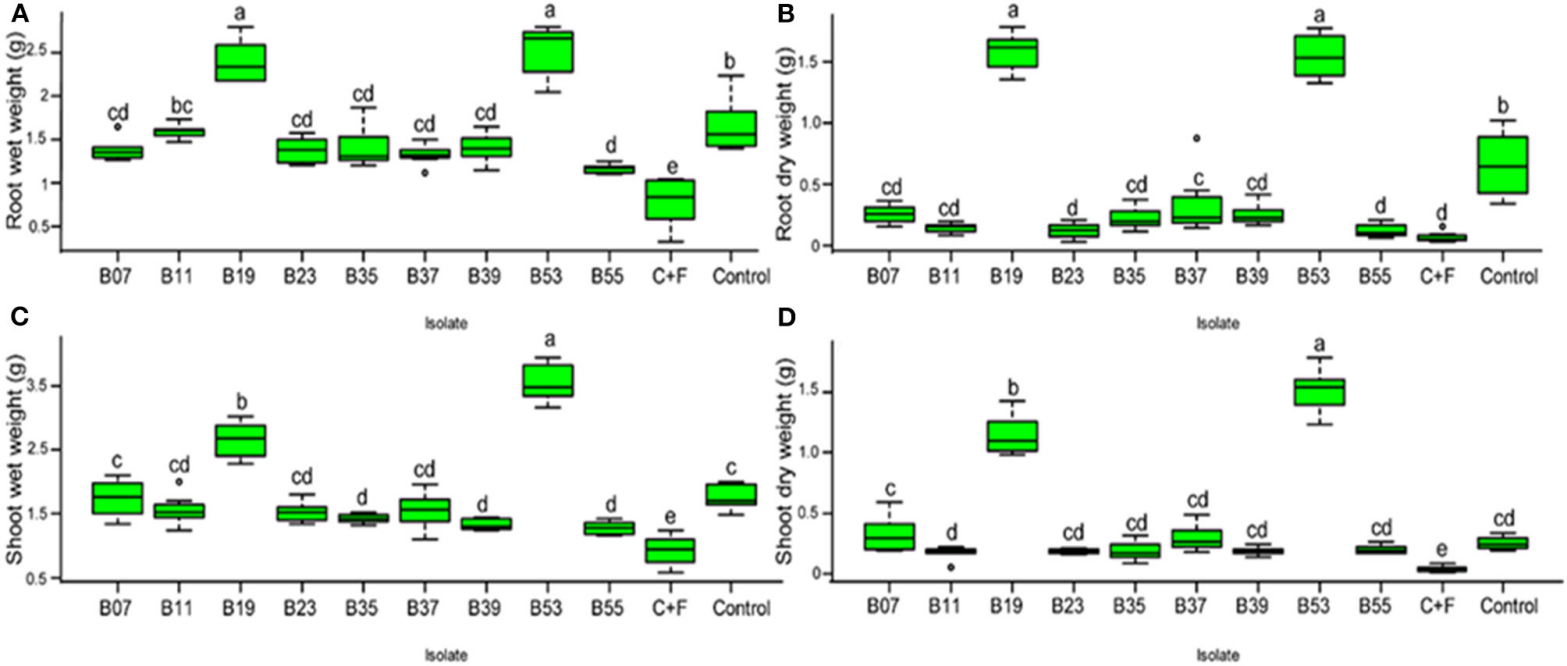
**(A–D)** Box plot represent the effect of bacterial endophytic inoculation on bean seedlings in the presence of *Fusarium solani* on the fresh and dry weights of the roots t and shoot. C+F represents a control with the growth of seedlings without endophyte in the presence of fungal pathogen while control represents seedling growth without pathogen or endophyte in a sterilized soil. Bars represent SE of the mean. Treatments with different letters are significantly different from each other (ANOVA test followed by SNK's *post-hoc* test, *P* < 0.05).

To confirm biocontrol activities exhibited by the bacterial endophytes on a dual plate assay and their effect on *F. solani* spores, a pot experiment was set up in a greenhouse. Bean seeds were bacterialized with the 9 selected endophytic bacteria and non-inoculated beans as controls. After 30 days of growth, treatment of bean seeds with bacterial endophytes significantly reduced the disease severity rating (Figure 5A) and the percentage of disease incidence (Figure 5B) when compared to the controls. Isolates, B53 and B19 did not show any symptoms of the disease at the end of the experiment and presented a well-developed root structure (Figure 5C). On the other hand, the treatment of bean seeds with B55 resulted in necrosis in the whole root system, yellowing, and eventual death of the seedlings similar to the control in fungus-infested soil (Figure 5C). Isolates B11, B07, B35, B37 and B23 indicated stagnated growth with poor and infected rooting system (Figure 5C). The first true leaves of the seedlings had turned yellow and easily fell off while washing the excess soil around the roots. Conversely, bacterial endophytes significantly affected the root wet weight (Figure 6A; F_10−77_ = 133.6, *P* < 0.0001), root dry weight (Figure 6B; F_10−77_ = 52.44, *P* < 0.0001), shoot wet weight (Figure 6C; F_10−77_ = 95.01, *P* < 0.0001), and shoot dry weight (Figure 6D; F_10−77_ = 184.3, *P* < 0.0001). Isolates, B19 and B53 significantly increased the wet and dry weights of the roots and shoots.

## Discussion

Lake Bogoria, one of Kenya's rift valley lakes is characterized by high pH and high salt deposits, occasional droughts causing precipitation of salts, and flooding during the rainy season that creates different hydrological cycles and a unique and changing environment especially for the shrub vegetation growing off the lake shore. The Lake has undergone significant hydrological changes in the last 10,000 years (Olago et al., 2009) and is still experiencing such changes in periods of extreme drought and flooding which have been on-going for the last 10 years. The vegetation thus has to adapt to these changes (Harper et al., 2003) and one of the known mechanisms of adaptation is through the recruitment of beneficial microorganisms that provide a selective advantage to the plants (Bulgarelli et al., 2013). These microorganisms are a storehouse of bioactive metabolites which help the plants tolerate both biotic and abiotic stresses (Fadiji and Babalola, 2020). Selim et al. (2012) hypothesized that plants in unique ecological niches may contain endophytes with vast metabolic ability for applications in various industries. Endophytic bacteria isolated from arid land plants by Asaf et al. (2017) were able to produce a variety of plant phytohormones and enhance the growth of soybean in a controlled environment. Similarly, Ali A. H. et al. (2018) demonstrated the ability of a thermophilic fungal endophyte isolated from a hot desert plant to confer heat stress tolerance to cucumbers under field conditions. The present study therefore sought to explore the potential of bacterial endophytes from shrubs along the draw-down zone of Lake Bogoria for the biocontrol of bean root rot pathogen, *F. solani*.

Identification of the isolates based on their 16S rRNA gene sequence revealed a bias in the isolation of the genus, *Bacillus*. The tendency to isolate the endophytic Bacillus species was previously noted from a variety of plants (Ali A. et al., 2017; Mohamad et al., 2020; Sendi et al., 2020). Isolation of endophytic bacteria from two mangrove systems in Brazil by Castro et al. (2014) also resulted in *Bacilli* as the most isolated genera followed by Gamma Proteobacteria, coinciding with our results. The rhizosphere is known to be the main source of endophytic microbiota (Andreote et al., 2014) and this could be the driving factor to this bias. Though there is limited information on rhizosphere microorganism from Lake Bogoria, other studies have indicated a similar abundance of the genera, *Bacillus* from both rhizosphere soil and plant endophytic bacteria. Besset-Manzoni et al. (2019) generated a culturable soil rhizosphere bacterial library, which resulted in *Bacillus* and *Pseudomonas* as the two most abundant genera. These genera have previously been isolated as endophytes. Their functional roles in plant growth promotion (Devi et al., 2017), biocontrol activities (Bendaha and Belaouni, 2019; Macedo-Raygoza et al., 2019), plant heat stress alleviation (Khan et al., 2020), and bioremediation (Wu et al., 2018) have been established.

Production of hydrolytic enzymes, antimicrobial metabolites, and plant phytohormones are important traits associated with most endophytic microorganisms (Khan et al., 2016; Potshangbam et al., 2017). Hydrolytic enzymes are important in the endophytic lifestyle as they are used by the microorganism to gain entry into the plant and in the control of phytopathogens and in eliciting stress tolerance. Some enzymes isolated from the endophytic bacteria include cellulases, proteases, lipases, pectinases, chitinases, and endoglucanases (Castro et al., 2014). Studies by Khan et al. (2018) correlated the antifungal activity of *Bacillus simplex* and *B. subtilis* against *Fusarium oxysporum f. sp. Conglutinans* and *F. solani* to the production of hydrolytic enzymes which resulted in fungal hyphal thinning and reduced disease development. Our results reveal similar activities with the isolates that showed delayed or inhibition of spore germination and high mycelial inhibition producing at least two of the enzymes tested. Phytohormones are important in the regulation of plant growth and development. Indole-3-acetic acid is an auxin found both in the plants and microorganisms and plays a major role in plant cell differentiation, in pathogenesis, and in the promotion of plant growth and survival under stress conditions (Egamberdieva, 2009; Vurukonda et al., 2016). One of the objectives of this study was to screen the endophyte collection for beneficial traits including the production of enzymes, phosphate solubilization, and production of hydrocyanic acid *in vitro*. A majority of the tested isolates were positive for plant growth promoting traits and enzymes, a trend also noted in other studies (Etesami et al., 2015; Mohamad et al., 2020).

From the results of the dual culture assay, a second set of *in vitro* antagonistic test was conducted to assess the effect of select bacterial cells from the spores of *F. solani* in co-cultures. Co-culturing of microorganisms provides cell to cell interactions that have been reported to facilitate the activation of cryptic pathways (Serrano et al., 2017), and hence the production of secondary metabolites that are not produced in axenic cultures that could potentially have antimicrobial activities (Bertrand et al., 2013). To test this concept on our isolates, we set up a cell spore direct interaction assay with each of the nine endophytic bacterial isolates with *F. solani* spores and incubated them overnight on a thin layer of PDA. The direct interaction of *F. solani* and the bacterial cells resulted in complete inhibition of germination of fungal spores by B53 and B19 (Figure 4). This further confirms the potential of the two isolates in the biocontrol of the test fungi. Fungal spores incubated with B39 and B07 showed delayed spore germination after an overnight incubation, with short germ tubes just starting to emerge. However, the two isolates did not control the fungus in planta, indicating loss of activity *in vivo*. Various studies on antagonist effects of two different strains through direct contact have been undertaken. Nogueira et al. (2019), while studying the effect of interaction between *Klebsiella pneumoniae* and *Aspergillus fumigatus*, confirmed that antagonism and inhibition of fungal spore germination were dependent on the direct contact of the two microorganisms that also affected biofilm formation. Further, Toghueo et al. (2016) indicated that the antagonistic activity of an endophytic *Trichoderma* spp. against *F. solani* in a spore–spore confrontation assay was concentration-dependent. In a natural environment, *Fusarium* spp. exist in both mycelia and clamydospores which infect crops when the conditions are favorable. Therefore, screening for the inhibition of mycelial and spore germination is an important factor to consider in the selection of an effective biocontrol agent in the field.

*F. solani* is a soil-borne pathogen which persists in soil after crop harvest until conditions are favorable and another crop is planted (Naseri, 2014) and it is difficult to control it. Some of the methods employed in the control include agronomic practices which do not offer effective control in addition to diminishing the availability of land. Thus, a better alternative is the use of biocontrol agents, which are mostly formulated for application as seed coating or sprays after seed and seedling germination (Bailly et al., 2020). However, the presence of fungal pathogens in soil significantly reduces the percentage of the germination of seeds (Botelho et al., 2013); therefore there is need to protect seeds and seedlings before planting. Though not widely supported in the application of biocontrols (Bailly et al., 2020), seed and seedling treatment with antifungal microbial based biocontrol agents can limit the invasion of the pathogen and enhance the germination rate. Seed coating with microbial biocontrols provides an immediate avenue for the colonization of both the roots and shoots as they emerge, offering protection against root pests and pathogens (O'Callaghan, 2016). Pereira et al. (2011) showed that seed treatment with *Bacillus amyloliquefaciens* reduced *Fusarium verticillioides* in maize roots. Seed soaking by Mangmang et al. (2015) resulted in enhanced germination value, root and shoot growth, vigorous and more consistent beneficial effects on tomatoes as compared to the drenching method. In our experiments, both seed soaking and drenching methods of endophyte delivery were used. The two methods showed significantly different colonization rates, with drenching method indicating a higher re-isolation titer than the seed soaking method. However, due to the inhibitory activity of *F. solani* in the germination of seeds, seed soaking method for endophyte delivery was chosen in green house experiments.

The mode of action of biocontrol agents *in vivo* is diverse and include competition for nutrients with the pathogen, production of antagonistic secondary metabolites, induction of systemic resistance, and priming the defense system of the host plant. Some of the metabolites involved in the biocontrol have been well characterized and documented (Khan et al., 2018; Köhl et al., 2019). Previous studies by López-Bucio et al. (2007) revealed the enhancement of bean root system by a *Bacillus megaterium* isolate through IAA and ethylene independent mechanisms. Association of plants with IAA producing microorganisms results in the elevation of IAA in the host plant (Spaepen et al., 2007), thereby stimulating the anti-oxidant system of the plant in response to stress conditions (Xia et al., 2015). Our findings corroborate with those of Mannaa et al. (2017) and Dahmani et al. (2020), who indicated that *B. megaterium* produced volatile metabolites with anti-fungal and growth promoting activities. Bendaha and Belaouni (2019) too, reported significant protection against *Fusarium oxysporum* f. sp. *radicis lycopersi* and growth enhancement in tomato seedlings using *Enterobacter hormaechei* subsp. *Steigerwaltii* isolate EB8D. However, in some experiments, *in vitro* antagonistic activities do not always correlate with biocontrol in planta (Besset-Manzoni et al., 2019). This was observed in B07 which had a considerably higher mycelial inhibition (49.8%) in dual culture assay and indicated delayed emergence of germ tubes from the spores in co-cultures, but did not show considerable fungal control in the pot experiment. Association of microorganisms with plants can lead to either gain of function or loss of function by the microorganism. Vandernkuornhuyse et al. (2015) hypothesized that plant-associated microorganisms are a consequence of adaptations and adjustments to environmental stress. These could probably explain the loss of antifungal activity *in vivo* by some isolates, and therefore predict other beneficial traits that could be associated with the bacterial endophytes from this study which were not screened.

In our selection, two isolates of *B. subtilis* were included in the screening (B11—*B. subtilis* subsp. *spezizenii* and B07—*B. subtilis*). Species of *B. subtilis* have been widely used in various industries including agriculture as biocontrol against plant pathogens and other industrial enzyme production processes (Su et al., 2020). However, our results indicate low levels of been rot protection against *F. solani* by the two *B. subtilis* isolates tested despite a considerable *in vitro* antagonism. In addition to industrial applications, the species has been implicated in plant adaptations to stress, a function that was noted by Abd-Allah et al. (2018) after inoculation of chickpea with endophytic *B. subtilis* isolate, BERA 71 under saline conditions which resulted in the modulation of the antioxidant system in the plants, reduction of reactive oxygen species (ROS), and significantly enhanced the growth of chickpeas. Association of microorganisms with plants has been noted as functionally driven (Wemheuer et al., 2017), and hence, based on the conditions of our sampling site, we hypothesized that the two endophytic *B. subtilis* isolates, and the other isolates found to have weak inhibition of the tested fungal mycelia might have different functional roles and therefore proposed further screening especially on abiotic stress tolerance and the production of enzymes for other applications.

Several studies on the microbial populations from Lake Bogoria have focused on the microbial diversity in water, sediments, and soil, with a bias on archaeal diversity and applications in industrial enzyme production. Results from this study have indicated the potential application of endophytic bacteria associated with shrubs growing along the draw-down zone of Lake Bogoria, a niche that has not been explored in the research on the microorganisms of soda lakes in Kenya. We isolated diverse endophytic bacteria, some of which are human-opportunistic pathogens. For example, *Enterobacter hormaechei* subsp. *Xiangfangensis* has been implicated as a human pathogen and in this study, it has indicated a greater potential in the biocontrol of the bean root rot pathogen, *F. solani*, in addition to plant growth promotion. Several other *Enterobacter* strains have been isolated from environmental samples and others as endophytes and have been shown to be beneficial to plants with growth promotion and biocontrol activities against phytopathogenic fungi (Ullah et al., 2017; Bendaha and Belaouni, 2019; Macedo-Raygoza et al., 2019; Przemieniecki et al., 2019). The biocontrol and growth-promoting ability of endophytic *Enterobacter hormaechei* is associated with the presence of several gene clusters in the genome, responsible for nitrogen fixation, phytohormone production, and transcriptional regulation, which are important traits for survival in diverse environments and plant endophyte interaction (Ren et al., 2010). Other strains of bacterial opportunistic and enteric human pathogens have been isolated as endophytes from various plants from diverse environments and vegetables (Lim et al., 2014; Nithya and Babu, 2017; Etminani and Harighi, 2018). However, Eberl and Vandamme (2016) noted that based on the source of the *Burkholderia* isolate, phylogenetic classification does not always infer human pathogenicity of a strain, and therefore a detailed genetic analysis is important. In another study, further genetic analysis of *Burkholderia* species grouped the pathogenic and plant associated strains in different clades, with the plant beneficial endophytic species lacking the virulence-associated loci necessary for human pathogenesis (Angus et al., 2014). In addition, Kandel et al. (2017), through a comparative based genomic analysis of *Burkholderia* species, found that endophytic *Burkhoderia* strains lacked key mammalian pathogenesis-related gene clusters in their secretion system and recommended their use in agricultural systems. We therefore recommend further studies to establish if these strains of endophytic *Enterobacter hormaechei* subsp. *Xiangfangensis* and *B. megaterium* contain human pathogenic properties. Further experiments to determine the endophytic localization of the bacteria in planta using fluorescent labeling and staining are necessary. In addition, further research should focus on the effectiveness of the two isolates in non-sterile soil and under field conditions.

## Conclusion

*Bacillus megaterium* isolated from the leaves of *Boeravia erecta* and *Enterobacter hormaechei* subsp., *Xiangfangensis* isolated from the roots of *Abutilon frutcosum* significantly reduced the effect of *F. solani* and increased the shoot and root weight of the bean plants. *In vitro*, the isolates produced IAA, solubilized phosphate, and the enzymes, catalase and amylase which are important stress-mitigation features. The isolates also inhibited mycelial and spore germination *in vitro*, further confirming their ability to control *F. solani*. However, further experimental studies are required in future to better understand the efficacy of the two isolates under non-sterile soil and field conditions, including the genetic mechanisms involved in the synergistic interaction of the plants and the microorganisms. More genetic studies are necessary to determine if the endophytic *Enterobacter hormaechei* subsp., *Xiangfangensis* contain any human pathogenesis-related genes.

## Data Availability Statement

The datasets presented in this study can be found in online repositories. The names of the repository/repositories and accession number(s) can be found below: https://www.ncbi.nlm.nih.gov/genbank/, GeneBank OK284374 - OK284406.

## Author Contributions

PM, VW, JO, and HB conceived and designed the experiments. PM performed the experiments with the guidance from VW, JO, HB, and EK and wrote the manuscript with the guidance and input from VW, HB, JO, EK, and SB. SB and PM analyzed the data. All authors have read, edited, and approved the final manuscript.

## Funding

PM was financially supported through a funding from the National Research Fund to undertake the research activities. The research activities were approved by the Kenya Wildlife Service under research Authorization Ref. KWS/BRM/5001 and NACOSTI Research Permit Number NACOSTI/P/17/22929/14802.

## Conflict of Interest

VW and EK were employed by Bioline Agrosciences—Dudutech IPM Limited. The remaining authors declare that the research was conducted in the absence of any commercial or financial relationships that could be construed as a potential conflict of interest.

## Publisher's Note

All claims expressed in this article are solely those of the authors and do not necessarily represent those of their affiliated organizations, or those of the publisher, the editors and the reviewers. Any product that may be evaluated in this article, or claim that may be made by its manufacturer, is not guaranteed or endorsed by the publisher.

## Supplementary Material

Supplementary Figure 1 IAA production by bacterial endophytes.

Supplementary Table 1 List of endophytic bacteria isolated in this study and closest relatives in gene bank.
