# Supplementary figures and images for: Culturable Bacterial Endophytes Associated With Shrubs Growing Along the Draw-Down Zone of Lake Bogoria, Kenya: Assessment of Antifungal Potential Against Fusarium solani and Induction of Bean Root Rot Protection
Source: Front Plant Sci. 2022 Feb 9;12:796847. doi: 10.3389/fpls.2021.796847 (PMC8864308; doi:10.3389/fpls.2021.796847)

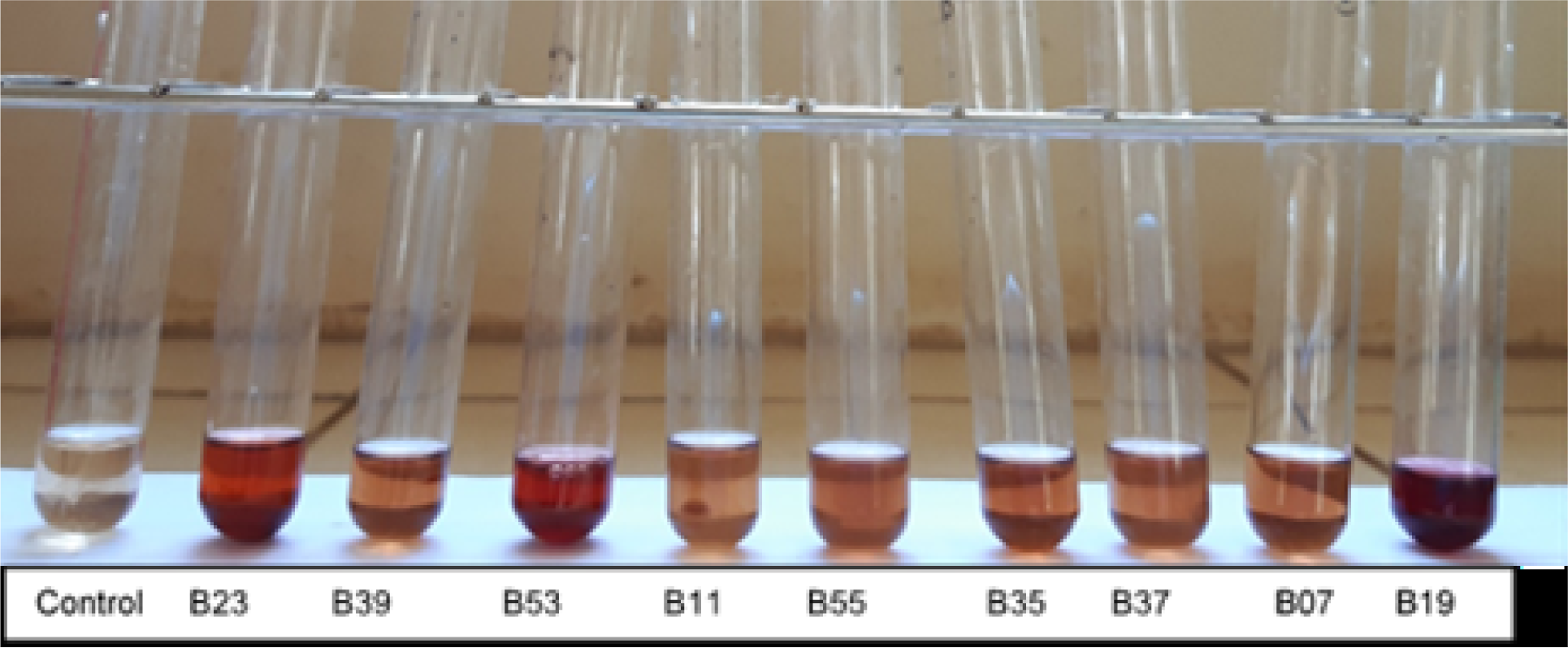

Supplement: Supplementary Figure 1 — IAA production by bacterial endophytes. [file Image_1.tif]
