# Supplementary material for: Culturable Bacterial Endophytes Associated With Shrubs Growing Along the Draw-Down Zone of Lake Bogoria, Kenya: Assessment of Antifungal Potential Against Fusarium solani and Induction of Bean Root Rot Protection
Source: Front Plant Sci. 2022 Feb 9;12:796847. doi: 10.3389/fpls.2021.796847 (PMC8864308; doi:10.3389/fpls.2021.796847)
Supplement: Supplementary Table 1 — List of endophytic bacteria isolated in this study and closest relatives in gene bank. [file Table_1.pdf]

Supplementary Table 1: List of endophytic bacteria isolated in this study and closest relatives in gene bank

Isolate code	Plant part	Name of source plant	Closest relative	Accession no.	% similarity
B01	Stem	*Solanum incanum*	*Bacillus cereus*	OK284403	100
B02	Root	*Abutilon fruticosum*	*Bacillus subtilis*	OK284374	99.78
B03	Leaves	*Boerhavia erecta*	*Bacillus haynesii*	OK284378	99.7
B04	Stem	*Solanum incanum*	*Bacillus cereus*	OK284402	100
B07	Root	*Solanum incanum*	*Bacillus subtilis*	OK284377	99.79
B08	Root	*Tephrosia uniflora*	*Pseudomonas aeruginosa*	OK284401	99.92
B11	Leaves	*Boerhavia erecta*	*Bacillus subtilis subsp. spezizenii*	OK284400	100
B13	Leaves	*Acalypha fruitcosa*	*Bacillus cereus*	OK284375	99.57
B14	Leaves	*Acalypha fruitcosa*	*Pseudomonas luteola*	OK284376	99.77
B16	Stem	*Rhynchosia Spp.*	*Pseudomonas aeruginosa*	OK284399	100
B19	Root	*Abutilon fruticosum*	*Enterobacter hormaechei subsp. Xiangfangensis*	OK284398	99.85
B20	Seed	*Prosopis juliflora*	*Bacillus subtilis*	OK284397	100
B21	Stem	*Rhynchosia Spp.*	*Pseudomanas aeruginosa*	OK284381	99.33
B22	Stem	*Acalypha fruitcosa*	*Bacillus subtilis*	OK284396	99.92
B23	Stem	*Tephrosia uniflora*	*Bacillus australimaris*	OK284386	100
B30	Leaves	*Solanum incanum*	*Bacillus australimaris*	OK284384	99.78
B31	Leaves	*Solanum incanum*	*Bacillus haynesii*	OK284385	99.79
B32	Leaves	*Acalypha fruitcosa*	*Bacillus velezensis*	OK284406	98.87
B44	Root	*Abutilon fruticosum*	*Bacillus subtilis*	OK284391	99.85
B52	Stem	*Abutilon fruticosum*	*Bacillus velezensis*	OK284404	100
B35	Stem	*Solanum incanum*	*Bacillus velezensis*	OK284389	99.87
B36	Leaves	*Boerhavia erecta*	*Micrococcus terreus*	OK284395	99.7
B37	Stem	*Ruelia patula*	*Bacillus tequilensis*	OK284394	99.92
B39	Leaves	*Solanum incanum*	*Bacillus aerius*	OK284393	99.71
B42	Stem	*Ruelia patula*	*Bacillus paramycoides*	OK284392	99.93
B43	Stem	*Solanum incanum*	*Bacillus subtilis*	OK284387	100
B46	Root	*Rhynchosia Spp.*	*Bacillus halotolerans*	OK284382	98.01
B47	Stem	*Boerhavia erecta*	*Bacillus subtilis*	OK284379	99.83
B51	Root	*Rhynchosia Spp.*	*Bacillus velezensis*	OK284405	99.88
B53	Leaves	*Boerhavia erecta*	*Bacillus megaterium*	OK284388	99.93
B54	Stem	*Solanum incanum*	*Bacillus cereus*	OK284383	98.92

B55	Leaves	*Tephrosia uniflora*	*Alkalihalobacillus clausii*	OK284380	99.11
B56	Root	*Solanum incanum*	*Pseudomonas aeruginosa*	OK284390	99.93
